# Bisphenol B Exposure Disrupts Mouse Oocyte Meiotic Maturation *in vitro* Through Affecting Spindle Assembly and Chromosome Alignment

**DOI:** 10.3389/fcell.2020.616771

**Published:** 2020-12-17

**Authors:** Shou-Xin Zhang, Zhi-Ming Ding, Muhammad Jamil Ahmad, Yong-Sheng Wang, Ze-Qun Duan, Yi-Liang Miao, Jia-Jun Xiong, Li-Jun Huo

**Affiliations:** ^1^Key Laboratory of Agricultural Animal Genetics, Breeding and Reproduction, Education Ministry of China, College of Animal Science and Technology, Huazhong Agricultural University, Wuhan, China; ^2^Biochip Laboratory, The Affiliated Yantai Yuhuangding Hospital of Qingdao University, Yantai, China; ^3^Hubei Province’s Engineering Research Center in Buffalo Breeding and Products, Wuhan, China

**Keywords:** bisphenol B, spindle assembly, chromosome alignment, DNA damage, epigenetic modifications

## Abstract

Bisphenol B (BPB), a substitute of bisphenol A (BPA), is widely used in the polycarbonate plastic and resins production. However, BPB proved to be not a safe alternative to BPA, and as an endocrine disruptor, it can harm the health of humans and animals. In the present study, we explored the effects of BPB on mouse oocyte meiotic maturation *in vitro*. We found that 150 μM of BPB significantly compromised the first polar body extrusion (PBE) and disrupted the cell cycle progression with meiotic arrest. The spindle assembly and chromosome alignment were disordered after BPB exposure, which was further demonstrated by the aberrant localization of p-MAPK. Also, BPB exposure increased the acetylation levels of α-tubulin. As a result, the spindle assemble checkpoint (SAC) was continuously provoked, contributing to meiotic arrest. We further demonstrated that BPB severely induced DNA damage, but the ROS and ATP production were not altered. Furthermore, the epigenetic modifications were changed after BPB exposure, as indicated by increased K3K9me3 and H3K27me3 levels. Besides, the pattern of estrogen receptor α (ERα) dynamics was disrupted with a mass gathering on the spindle in BPB-exposed oocytes. Our collective results indicated that exposure to BPB compromised meiotic maturation and damaged oocyte quality by affecting spindle assembly and chromosome alignment, acetylation of α-tubulin, DNA damage, epigenetic modifications, and ERα dynamics in mouse oocytes.

## Introduction

Endocrine disrupting chemicals (EDCs), a group of exogenous substances or compound mixtures, can interfere with hormone action in the body and disrupt endocrine function that harms human and animal health (WHO/IPCS, 2002). Bisphenol A (BPA), a common manufacturing chemical in polycarbonate plastics is being widely used in industrial production, is one of the most abundant EDCs (Usman and Ahmad, 2016). Widespread use of BPA-containing products results in ubiquitous BPA exposure leading to a persistent and elevated environmental level. Bio-monitoring measurements of BPA in human serum, urine, hair, semen, and breast milk have revealed widespread exposure to BPA in the human population (Vandenberg et al., 2007; Usman and Ahmad, 2016). As an endocrine disruptor, BPA has been attracting increasing attentions, and many researchers are digging to explore how the endocrine system disrupted by bisphenol B (BPB) (Weatherly and Gosse, 2017; Ma et al., 2019a; Yang et al., 2019). Concerning the public health issue of reproductive disorder, the involvement of EDCs is suspected (Ma et al., 2019b). It has been reported that BPA has adverse effects on reproductive systems (Castellini et al., 2020). In males, BPA exposure decreases testosterone levels, affects sperm production and quality, and increases sperm DNA damage (Wisniewski et al., 2015; Lombó et al., 2019). BPA may influence the cyclicity negatively in females and cause ovarian and uterine dysfunction, including steroidogenesis, follicular formation, and oogenesis (Ziv-Gal and Flaws, 2016; Pivonello et al., 2020). Due to its reproductive toxicity and endocrine disrupting properties, the use of BPA is restricted.

The public concern and restrictions on BPA promote the development of alternative substances to replace BPA. BPB, as a substitute of BPA, is widely used in the production of polycarbonate plastic and resins (Cunha and Fernandes, 2010). Different food items such as canned foods, drinks, meat, peeled tomatoes, beverage, and milk for infants have been detected with BPB (Grumetto et al., 2008; Cunha et al., 2011; Cunha et al., 2012). BPB is resistant to aerobic and anaerobic bio-degradation, which makes it more prone to bio-accumulate in nature (Chen et al., 2016; Usman and Ahmad, 2016). BPB has been detected in various samples from humans such as sera and urine (Cobellis et al., 2009; Cunha and Fernandes, 2010). BPB is structurally similar to BPA with analogous physicochemical properties, and it was reported to affect male reproductive system negatively. Both acute and subacute exposures of adolescent male mice to BPB adversely impact tests and morphology of the sperm and their function (Ullah et al., 2018a, b; Ikhlas and Ahmad, 2020). Adult rats subjected to 50 mg/kg/day of BPB for 30 days have experienced sperm cell DNA damage (Ullah et al., 2018a). Ovaries in BPB treated female rats indicated adverse morphological and histopathological alterations, including a notable decrease in antral follicles and corpus luteum and the rise in atretic and cystic follicles (Ijaz et al., 2020). A relevant study has reported dose-dependently impaired reproductive functions in male and female zebrafish exposed to BPB, evidenced by a lower number of eggs laid, and a smaller hatching rate and embryo survival, reaching statistical significance in the 1 mg/L group (Yang et al., 2017). Despite the above experiments, the effects of BPB on reproductive function of animals and humans have been poorly studied.

Female fertility is greatly affected by the oocytes’ quality, which is linked to clear and accurate meiotic division. The meiotic maturation process involves distinct spindle organization and chromosome alignment and segregation, which is regulated by the microtubule organizing center (MTOC) (Bennabi et al., 2016; Mogessie et al., 2018). To avoid false chromosome segregation, the continuous activation of SAC works as a checkpoint, which inhibits the onset of anaphase till the attachment of all chromosomes to spindle microtubules and align at the metaphase plate (Lara-Gonzalez et al., 2012). BPA has been reported to disrupt the oocyte maturation process, affect cytoskeletal dynamics, induce oxidative stress and DNA damage, alter the epigenetic modifications, and even culminate in oocyte apoptosis (Wang et al., 2016). Other bisphenol substitutes, like bisphenol S (Žalmanová et al., 2017), bisphenol AF (Ding et al., 2017), and Fluorene-9-bisphenol (Jiao et al., 2019), were also demonstrated to inhibit mouse oocyte maturation and deprave oocyte quality. Nevertheless, related to oocytes maturation, the effects of BPB on oocyte maturation has never been addressed, and it remains to explore whether BPB have the same effects like BPA.

The objective of this study was to evaluate the effects of BPA on mouse oocyte maturation and its related mechanisms *in vitro*, and by evaluating the ratio of PBE, ROS levels, DNA damage, spindle morphology, chromosome alignment and segregation, ER, and epigenetic modifications. Our study could determine the novel toxicological mechanisms of BPB on oocyte maturation and create awareness about the safety of BPA substitutes.

## Materials and Methods

### Animals and Ethics Statement

In this study, we used female of Kunming mice (3–4 weeks). The experimental mice were bought locally and kept in the Laboratory Animal Center of Huazhong Agricultural University under 12 h light/dark cycle. These mice received food and water *ad libitum*. All the experiments were performed according to the rules set out by the Huazhong Agricultural University Animal Care and Use Committee (HZAUSW-2017-005).

### Antibodies and Chemicals

Primary antibodies detailed information including host species, vendor, catalog number, and working concentration are given in Table 1. BPB was purchased from Aladdin (Shanghai, China); Dihydroethidium was obtained from Beyotime (Beijing, China); CellTiter-Glo ATP Assay Kit was purchased from Promega (Madison, Wisconsin). Sigma (St. Louis, MO, United States) supported all remaining reagents, except for any other variable.

**TABLE 1:** Detailed information of antibodies.

Antibody	Host spices	Vendor	Catalog no.	Working dilution
				**IF**
α-tubulin-FITC	Mouse	Sigma	F2168	1:100
H3K9me3	Rabbit	Affinity	DF6938	1:100
H3K27me3	Rabbit	Affinity	DF6941	1:100
Estrogen receptor alpha	Rabbit	Abcam	ab32063	1:100
BubR1	Rabbit	Abcam	ab254326	1:50
Alpha tubulin (acetyl K40)	Mouse	Abcam	ab179484	1:100
Phospho-p44/42 MAPK	Rabbit	Cell Signaling Technology	4370T	1:100
Cy3-conjugated anti-rabbit IgG	Goat	Boster	BA1032	1:100

### Oocytes Collection and Culturing

Cumulus oocytes complexes were obtained from ovaries of Female K. M aged 3–4 weeks, primed with pregnant mare serum gonadotropins for 48 h, by manual puncturing of antral ovarian follicles. Cumulus cells and oocytes were separated by recurrent pipetting. For the persistence of GV-stage, the oocytes were retrieved in preheated (37°C) DMEM/F12 medium with IBMX (50 μM). To stimulate the meiotic maturation, oocytes (GV) were washed out of IBMX and cultured in M16 medium at 37°C in a humidified atmosphere of CO_2_ (5%).

We used *in vitro* culture model to check the effect of BPB on oocyte meiotic maturation and oocyte quality. Therefore, we used a much higher concentration of BPB in this manuscript, to check what concentration could be toxic to oocyte, and what are the defects induced by BPB, and related regulatory pathways. DMSO dissolved BPB was diluted with M16 medium to obtain the relevant concentrations (DMSO, <0.3%) of 0, 50, 100, 150, and 200 μM to study the possible effects on oocyte maturation *in vitro*. The first PBE (*in vitro* oocyte maturation sign) rate was observed. Though 150 μM BPB exposure significantly reduced PBE rate, however, a limited number of oocytes were capable to continue the meiosis, which could help to uncover the BPB molecular mechanism affecting the oocyte maturation. Hence, a concentration of 150 μM was selected to investigate the molecular mechanism.

### Immunofluorescence Staining

Firstly, oocytes were washed with PHEM solution (60 mM PIPES at pH 6.9, 25 mM HEPES, 10 mM EGTA, 2 mM MgCl2.7H2O). Next, oocytes (stage-specific) were fixed in PHEM solution (4% paraformaldehyde, 0.5% Triton X-100) for 45 min. Subsequently following blocking in PBS with 2% BSA and 0.05% Tween-20 at 25°C for 1 h, oocytes were incubated in the refrigerator overnight at 4°C with the primary antibodies mentioned (Table 1). Following primary antibody incubation, PBS plus Tween-20 (0.05%) was used to wash oocytes three times for 10 min each to incubate with the second antibody (Cy3-labeled goat anti-rabbit, Boster, 1:100) at 37°C for 1 h. For labeling DNA, at room temperature, oocytes were incubated with DAPI (1 μg/ml) in PBS for 10 min. Finally, DABCO was used to mount oocytes on glass slides for confocal laser scanning microscopy (Zeiss LSM 510 META, Carl Zeiss Imaging, Germany) equipped with an objective DIC. Plan-Apochromat 63/1.4 oil. Visualization of confocal images was made by subjecting these images to LSM. Image Browser software and Adobe Photoshop (Adobe Systems Inc., San Jose, CA, United States). Primary antibodies were replaced with non-immunized rabbit or goat IgG for negative control.

Dihydroethidium was used to determine the level of ROS. Before measuring fluorescence, the confocal microscope was adjusted to the same parameters. Each group of oocytes underwent the same immunostaining procedure for optimizing the acquired signals in oocytes from control and treatment groups. ImageJ software (NIH, United States.) or LSM. Image Browser software (Zeiss, Germany) was used to analyze the relative mean intensity of the fluorescence.

### Chromosome Spreading

Following treatment with Tyrode buffer (pH 2.5) at room temperature, oocytes detached from Zona pellucida were retrieved in M2. Retrieved oocytes were fixed on glass slides in a drop of 1% paraformaldehyde with Triton X-100 (0.155), 3 mM DTT. After air drying, slides were washed (PBS) and blocked (BSA, 2%). Next, following incubation (anti-BubR1 rabbit, 1:50, 4°C, overnight), and (CY3-conjugated anti-rabbit sheep antibody, 1:100, 37°C, 1 h), respectively, DAPI was used to counterstain the chromosomes and for confocal laser scanning microscopy.

### ATP Assessment

Relative ATP concentrations were determined using the kit method (CellTiter-Glo^®^ ATP Assay Kit) (Promega, Madison, WI, United States) as per manufacturer instructions. Briefly, control or treatment (BPB) oocytes were transferred (*n* = 30, each) into 96-well black culture plates (M16, 50 μl/well) and added a reagent (CellTiter-Glo^®^; 50 μl/well; 10 min, 25°C) for luminescent signal stability. Luminescence against ATP concentration was obtained by reading the Plates using EnSpire^®^ Multimode Reader (PerkinElmer, Waltham, MA, United States).

### Statistical Analysis

All the data were obtained from three independent experiments and documented (M ± SEM). Data were analyzed using the analysis software Graph-Pad Prism with paired samples *t*-test. The difference was considered statistically significant if *P*-values (0.05).

## Results

### BPB Exposure Compromised the Meiotic Maturation of Mouse Oocytes

**FIGURE 1:**
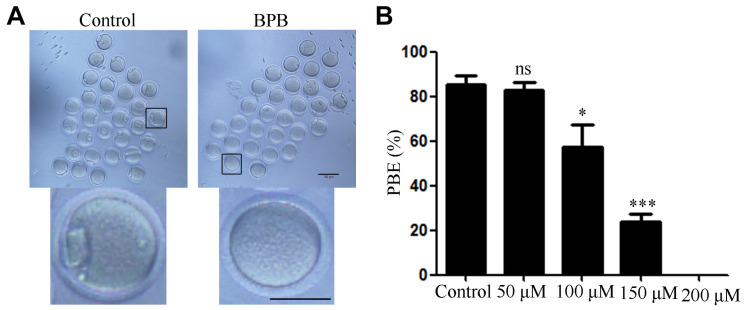
BPB exposure compromised the meiotic maturation of mouse oocytes. **(A)** Images depicting oocyte maturation with BPB (0, 50, 100, 150, 200 μM) exposure for 14 h. Bar,100 μm; Bar, 100 μm (small graph). **(B)** Rate of oocytes that extruded the first PB in control and BPB-treated groups. Control, *n* = 117; BPB, *n* = 88. ^∗^Significantly different (*P* < 0.05); ^∗∗∗^Significantly different (*P* < 0.001) compared with the corresponding control.

We first examined the oocyte meiotic ability of BPB-exposed oocytes by calculation of the first PBE rate in oocytes exposed to different concentration of BPB (0, 50, 100, 150, and 200 μM) for 14 h. Results showed that BPB exposure compromised the oocyte meiotic maturation (Figure 1A). Statistical result showed that the PBE rate decreased in the BPB-exposed groups (100, 150, and 200 μM) and this decrease was significant for the 150 μM BPB group from 85% in controls to 66% in BPB-exposed oocyte (85.43 ± 3.9%, *n* = 117 vs. 23.77 ± 3.7%, *n* = 88; *P* < 0.001; Figure 1B). The use of 150 μM of BPB has led to a considerable reduction in PBE rates, but a small number of oocytes have been able to continue meiosis and maturation, which could help to investigate the BPB molecular mechanism which affects oocyte maturation. Hence, the concentration of 150 μM was chosen to uncover the molecular mechanism in the subsequent experiments.

### BPB Exposure Disturbed Cell Cycle Procession of Meiotic Maturation in Mouse Oocytes

**FIGURE 2:**
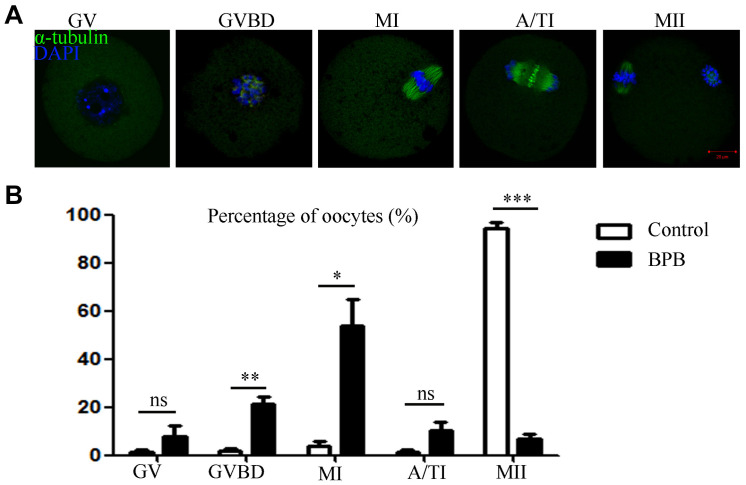
BPB exposure disturbed cell cycle procession of meiotic maturation in mouse oocytes. **(A)** Spindle and chromosome depiction during oocyte developmental stages (GV, GVBD and Pro-MI, MI, A/TI, and MII). α-tubulin, green; DNA, blue. Bar, 20 μm. **(B)** The cell cycle distribution was quantified in the control and BPB groups. Control, *n* = 90; BPB, *n* = 90. ^∗^Significantly different (*P* < 0.05); ^∗∗^Significantly different (*P* < 0.01); ^∗∗∗^Significantly different (*P* < 0.001).

Since BPB exposure resulted in the failure of meiotic maturation, the cell cycle progression was analyzed after 14 h of culture, grouped according to the developmental arrest at different meiotic stages (Figure 2A). As shown in Figure 2B, most of control oocytes reached MII stage, while most of BPB-exposed oocytes were still arrested at the GVBD or MI stage (GVBD: 2.000 ± 1.1%, *n* = 90 control vs. 21.20 ± 3.2, *n* = 90; *P* < 0.01; MI: 3.633 ± 2.1, *n* = 90 control vs. 53.87 ± 10.8, *n* = 90; *P* < 0.05). Notably, no significant difference was observed between the control group and the BPB-exposed group of GV phase and A/TI phase (GV: 1.200 ± 1.2, *n* = 90 control vs. 8.033 ± 4.1, *n* = 90; P > 0.05; A/TI: 1.200 ± 1.2, *n* = 90 control vs. 10.13 ± 3.5, *n* = 90; *P* > 0.05). These data demonstrated that BPB treatment significantly disturbed meiotic progression.

### BPB Exposure Affected Spindle Assembly and Chromosome Alignment in Mouse Oocytes

**FIGURE 3:**
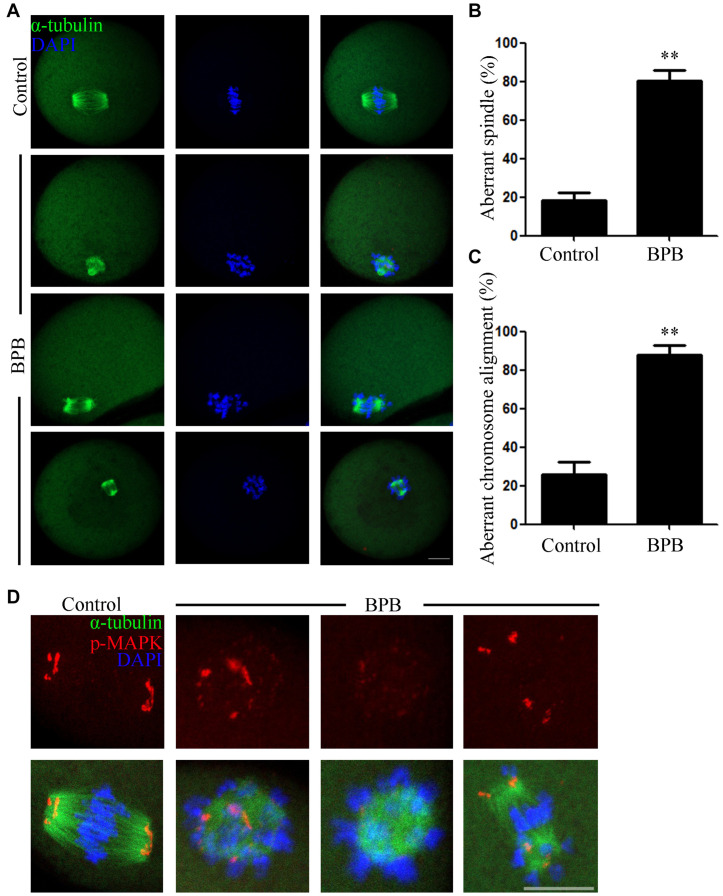
BPB exposure disturbed the MI spindle assembly and chromosome alignment. Only the oocytes that reach to GVBD at 2 h in the control and BPB treated group were cultured for 8 h for subsequent analysis. **(A)** Spindle morphology and chromosome alignment depiction in control and BPB-treated oocytes. α-tubulin, green; DNA, blue. Bar, 20 μm. **(B)** Aberrant spindle morphology rate following BPB exposure. Control, *n* = 92; BPB, *n* = 91. ^∗∗^Significantly different (*P* < 0.01). **(C)** Aberrant chromosomal alignment rate following BPB exposure. Control, *n* = 92; BPB, *n* = 91. ^∗∗^Significantly different (*P* < 0.01). **(D)** Depiction of p-MAPK position in control and BPB-exposed oocytes. p-MAPK, red; α-tubulin, green; DNA, blue. Bar, 20 μm.

Considering a meiotic arrest mediated by defective spindle morphology largely induced activation of SAC, we next examined the MI oocytes for spindle organization and chromosome alignment. Spindle structure was observed using an anti-α-tubulin-FITC antibody, and DAPI was used for visualization of chromosome alignment. The results displayed a typical barrel-shaped spindle apparatus in controlled oocytes, and the equatorial plate had well-aligned chromosomes. In contrast, abnormal spindle morphology and misaligned chromosomes were observed in BPB-treated oocytes (Figure 3A). Statistically, the rate of abnormal spindles and misaligned chromosomes were significantly increased from 18% in controls to 80% and from 25% in controls to 87%, respectively (abnormal spindles: 18.30 ± 4.3% control, *n* = 92 control vs. 80.10 ± 5.9%, *n* = 91; *P* < 0.01; misaligned chromosomes: 25.87 ± 6.3%, *n* = 92 control vs. 87.83 ± 4.9%, *n* = 91; *P* < 0.01; Figures 3B,C). Moreover, p-MAPK was examined to explain the mechanism for spindle defects following BPB exposure to the well-established component of MTOCs. In contrast to wild MI oocytes, where p-MAPK in spindle poles is canonically enriched, BPB-exposed oocytes show a severe distorted p-MAPK localization. BPB exposure resulted in the detachment of p-MAPK from spindle poles with scattered signals around the spindle, and some BPB-treated oocytes showed a precipitous decline in p-MAPK expression (Figure 3D), indicating the dysfunction of MTOCs. Moreover, the aberrant spindle assemble and chromosome alignment were found in MII oocytes after BPB treatment (aberrant spindle and chromosome: 17.81 ± 9.0%, *n* = 102 control vs. 65.34 ± 3.5%, *n* = 91; *P* < 0.01; Supplementary Figures 1A,B). Thus, these data suggested that BPB exposure disrupted meiotic spindle assembly and chromosome alignment, which contributed to meiotic failure after BPB treatment.

### BPB Exposure Changed the Acetylation Levels of α-Tubulin in Mouse Oocytes

**FIGURE 4:**
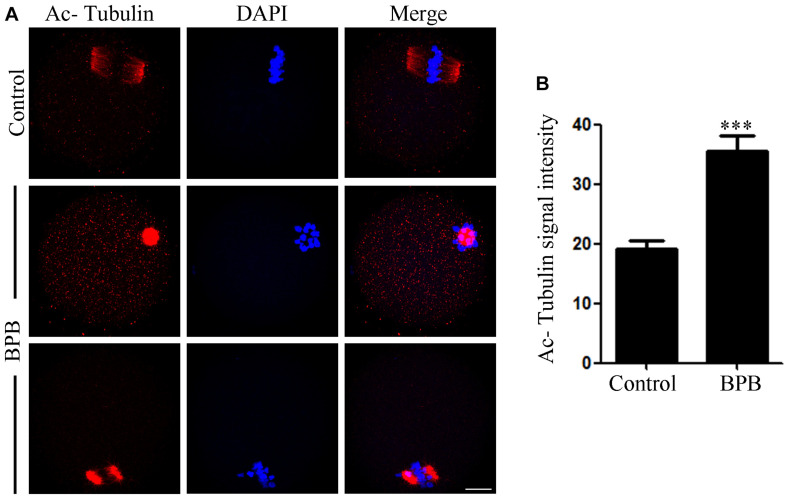
BPB exposure increased the acetylation level of α-tubulin. **(A)** Acetylated α-tubulin depiction in control and BPB-treated oocytes. Acetylated α-tubulin, red; DNA, blue. Bar, 20 μm. **(B)** Quantification of fluorescence intensity for acetylated α-tubulin in control, and BPB-exposed oocytes. Control, *n* = 34; BPB, *n* = 29. ^∗∗∗^Significantly different (*P* < 0.0001).

In tubulin, one of the most abundant non-histone proteins, lysine 40 of α-tubulin subunit is the site of acetylation (Zilberman et al., 2009). The microtubules stability in mouse oocytes is dependent on α-tubulin acetylation levels (Ling et al., 2018), and aberrant levels of α-tubulin acetylation may affect spindle assembly and the meiotic process. As spindle defects were confirmed, we then examined the acetylation status of microtubules in control and BPB-treated oocytes. Results from Figures 4A,B revealed that the BPB-exposed oocytes had a significantly higher level for tubulin acetylation compared to the control (19.17 ± 1.466, *n* = 34 control vs. 35.47 ± 2.570, *n* = 29; *P* < 0.0001).

### BPB Exposure Continuously Activated the SAC in Mouse Oocytes

**FIGURE 5:**
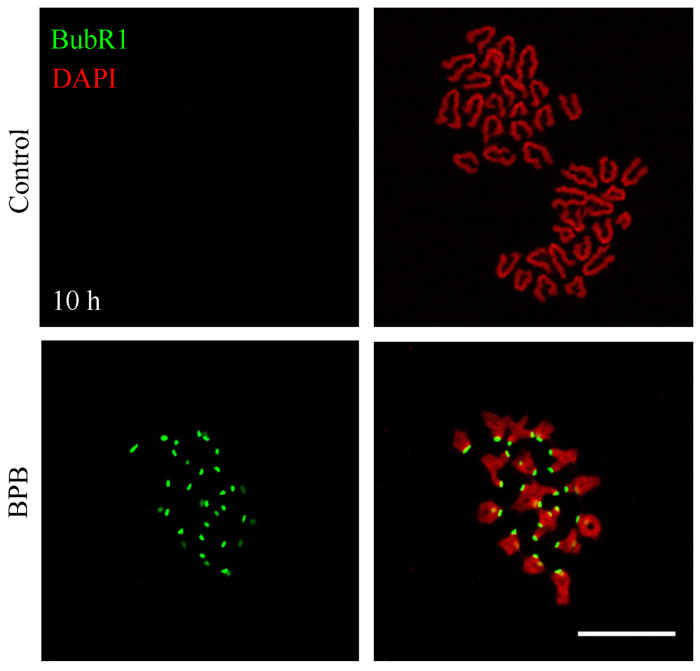
BPB exposure continuously activated the spindle assembly checkpoint. BubR1 depiction in control and BPB-exposed oocytes at ATI stage. BubR1, green. DNA, red. Bar, 20 μm.

Meiotic arrest and impaired spindle assembly in BPB-treated oocytes suggest that SAC might always be activated. To gain insight into this issue, BubR1, an integral component of SAC, was detected by chromosome spreading to indicate SAC activation at 10 h (A/TI) after BPB exposure. The control group did not show BubR1 signals, whereas a clear BubR1 signal was observed still in BPB exposed oocytes at the kinetochores (Figure 5), which indicates SAC activation.

### BPB Exposure Increased DNA Damage in Mouse Oocytes

**FIGURE 6:**
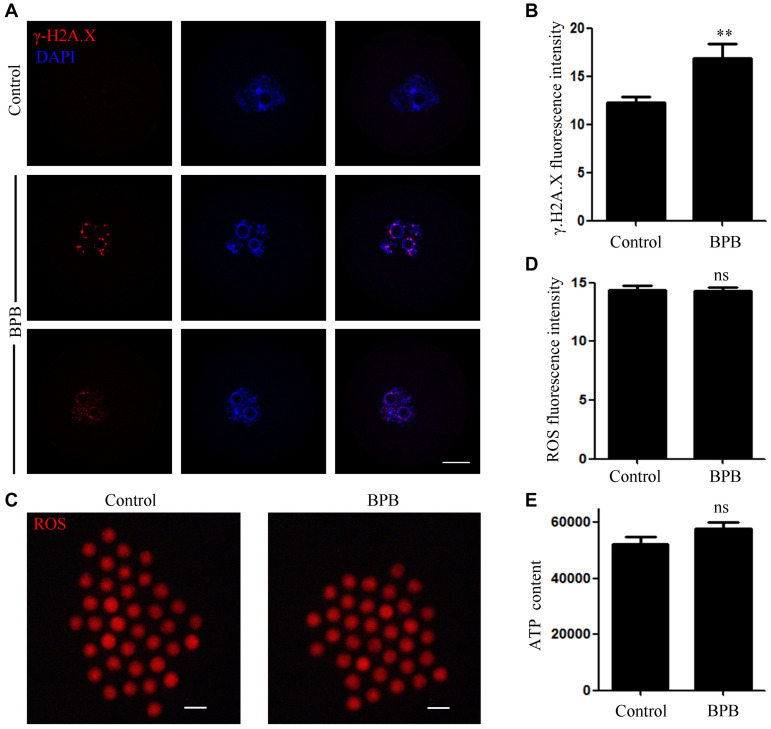
BPB exposure increased DNA damage in mouse oocytes. **(A)** Images depicting DNA damage in control and BPB-treated oocytes. γ.H2A.X, red; DNA, blue. Bar, 20 μm. **(B)** Quantitative analysis of the fluorescence intensity of γ.H2A.X in control and BPB-treated oocytes. Control, *n* = 33; BPB, *n* = 32. ^∗∗^Significantly different (*P* < 0.01). **(C)** ROS depiction in control and BPB-exposed oocytes. ROS, red. Bar, 100 μm. **(D)** ROS fluorescence intensity in control and BPB-exposed oocytes. Control, *n* = 37; BPB, *n* = 36. No significant difference (*P* > 0.05). **(E)** The ATP content in control and BPB-treated oocytes. Control, *n* = 30; BPB, *n* = 30. No Significantly (*P* > 0.05).

Considering that BPB exposure induced DNA damage in sperm cells, so we want to know whether BPB can also cause DNA damage in oocytes. DNA double strand breaks (DSBs) is one of the most common types of DNA damage. Therefore, the anti-γ.H2A.X antibody was used to indicate DNA damage in mouse oocytes. As shown in Figure 6A, bright γ.H2A.X foci prevalence in BPB-exposed oocytes inferred the induction of severe DNA damage. Quantitative analysis further demonstrated that the DNA damage level significantly increased after BPB treatment (12.23 ± 0.6193, *n* = 33 control vs. 16.88 ± 1.447, *n* = 32; *P* < 0.01; Figure 6B).

We then detected the ROS level in the BPB-exposed oocytes. Unexpectedly, no obvious change was observed between the BPB-exposed oocytes and the controls (Figure 6C), indicating that oxidative stress was not produced by BPB. Moreover, fluorescence intensity of ROS analysis further confirmed this conclusion (14.36 ± 0.3942, *n* = 37 control vs. 14.29 ± 0.3002, *n* = 36; *P* > 0.05; Figure 6D). Besides, the ATP content was also not altered after BPB exposure (52,220 ± 2688, *n* = 30 vs. 57,860 ± 2041, *n* = 30 control; *P* > 0.05; Figure 6E).

### BPB Exposure Altered the Levels of Histone H3K27me3 and H3K9me3

**FIGURE 7:**
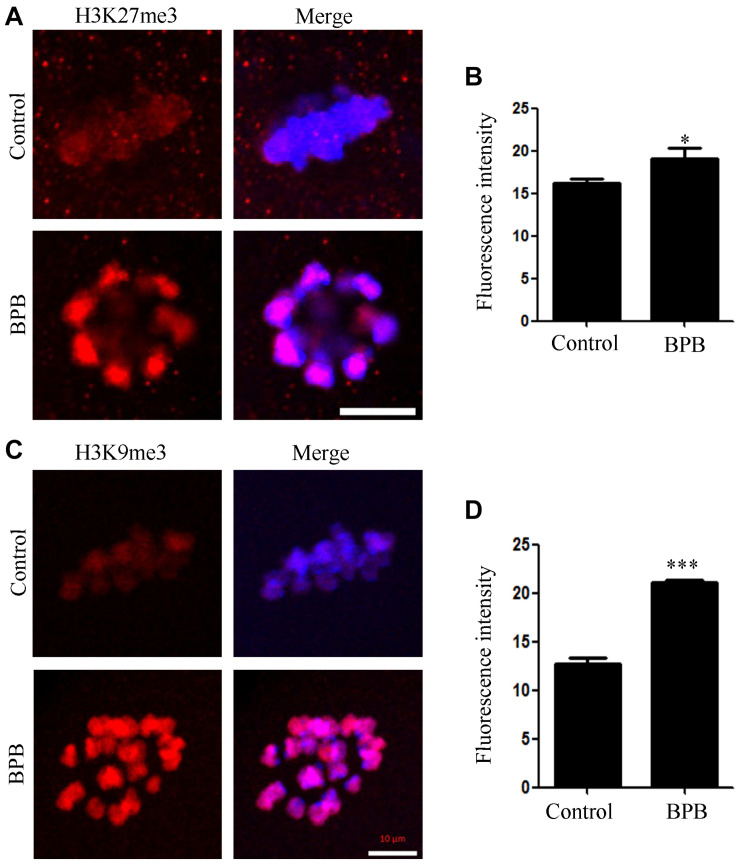
BPB exposure altered epigenetic modification in mouse oocytes. **(A)** Images depicting H3K27me3 in control and BPB-treated oocytes. H3K27me3, red. Bar, 10 μm. **(B)** H3K27me3 fluorescence intensity in control and BPB-exposed oocytes. Control, *n* = 29; BPB, *n* = 31. ^∗^Significantly different (*P* < 0.05). **(C)** Images depicting H3K9me3 in control and BPB-treated oocytes. H3K9me3, red. Bar, 10 μm. **(D)** H3Kme3 fluorescence intensity in control and BPB-exposed oocytes. Control, *n* = 30; BPB, *n* = 29. ^∗∗∗^Significantly different (*P* < 0.0001).

The effects of BPB exposure on epigenetic modification were evaluated by examining the H3K27me3 and H3K9me3 levels. The signals of H3K27me3 were significantly increased in the BPB-exposed oocytes than in the control group. Consistently, the fluorescence intensities of H3K9me3 were significantly higher in the BPB-exposed oocytes. Statistical analysis further confirmed that BPB affected the levels of histone H3K27me3 and H3K9me3 (H3K27me3: 16.16 ± 0.5889, *n* = 29 control vs. 19.11 ± 1.183, *n* = 31, *P* < 0.05; Figures 7A,B; H3K9me3: 12.68 ± 0.6714, *n* = 30 control vs. 21.10 ± 0.2463, *n* = 29, *P* < 0.0001; Figures 7C,D).

### BPB Exposure Disrupted the Localization Patterns of ERα

**FIGURE 8:**
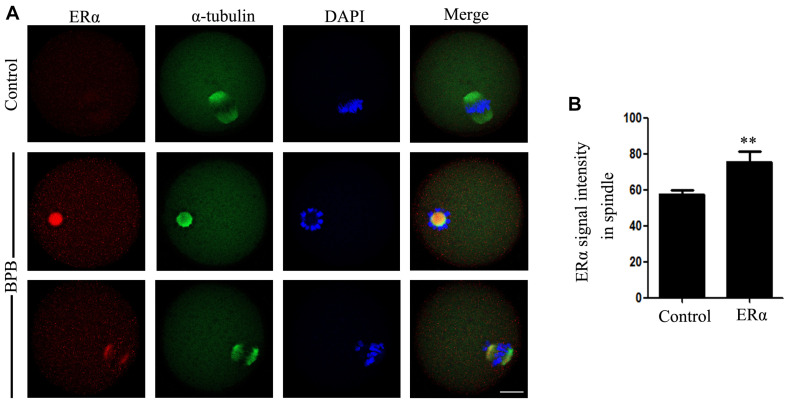
BPB exposure changed the distribution pattern of ERα. **(A)** ERα depiction in control and BPB-exposed. ERα, red; α-tubulin, green; DNA, blue. Bar, 20 μm. **(B)** ERα fluorescence intensity on the spindles in control and BPB-exposed oocytes. Control, *n* = 34; BPB, *n* = 33. ^∗∗^Significantly different (*P* < 0.01).

Since BPB was suggested to be competitively bound to ER of several species, including human and mouse (Blair et al., 2000; Sipes et al., 2013; Zhang et al., 2018), we finally tested the changes in estrogen receptor α (ERα) in controls and BPB-exposed oocytes. ERα signals in control-oocytes were diffused in the cytoplasm. At the same time, ERα had accumulated separately around the chromosome and showed a spindle-like pattern in oocytes exposed to BPB (Figure 8A). In addition, fluorescence intensity analysis showed that ERα signals were sharply increased in the BPB-exposed oocytes spindle in comparison to control oocytes (57.56 ± 2.209, *n* = 34 control vs. 5.54 ± 6.005, *n* = 33; *P* < 0.01; Figure 8B). Collectively, these data indicated that BPB exposure disrupted the localization patterns of ERα.

## Discussion

Human beings are exposed to EDCs through various channels throughout their life cycle. EDCs can be absorbed by the body, accumulated, and even bio-transformed into more toxic metabolites. The EDCs including BPA are widely distributed and have adverse effects, which increased the researchers’ attention toward it, and more and more studies aimed at explaining how these compounds affect the endocrine system (Cimmino et al., 2020). The safety of BPB as a substitute for BPA has also attracted people’s attention. Most of the assays considered BPB has the potency similar to or even greater than that of BPA. Adverse effects of BPB on fish reproduction and the male reproductive system in rodents has been reported, but the effects on the female are still obscure.

Endocrine disrupting chemicals can disrupt ovarian physiology resulting in undesirable reproductive effects such as estrogen deficiency, dysfunctional ovulation, premature ovarian failure, and even infertility. The line of evidence for BPB estrogenic activity has been obtained in various *in vivo* and *in vitro* experiments (Serra et al., 2019). Ovarian estrogens and estrogen/ER pathways are indispensable for the development and physiology of female organs and female reproduction in mammals (Wall et al., 2014). BPB is competitively bound to ER of several species, including human and mouse (Blair et al., 2000; Sipes et al., 2013; Zhang et al., 2018). ERs, including ERα and ERβ, belong to a large nuclear receptor superfamily and can function as the ligand-induced transcription factors (Hall and McDonnell, 2005). BPB was confirmed to express ERα through ER-regulated gene expression in human mammary MCF-7 cell lines (Rivas et al., 2002; Mesnage et al., 2017). In fish exposed from 5 μM of BPB, the expression of ERα and hepatic estrogen genes vitellogenin-1, choriogenin-L was significantly high (Yamaguchi et al., 2015). A dose-dependent higher RNA expression of ERα and ER-regulated cyp19a1b was observed in male zebrafish brain exposed for 21 days from 0.1 mg/L (Yang et al., 2017). Besides, ERα appears to play a significant role in major reproductive physiological functions in females (Lee et al., 2009). Our data displayed alteration in the distribution of ERα, which was significantly aggregated on the spindle in BPB treated mouse oocytes. During mitosis, ERα is located on the spindles for the alignment of chromosome and spindle dynamics (Zhang et al., 2015). Several EDCs have been reported affecting oocytes maturation through targeting ERs. In pig oocytes, bisphenol S enhances the ERα expression which affects the oocyte meiotic maturation negatively (Žalmanová et al., 2017). In our previous study, diethylstilbestrol was found to neutralize oocyte meiotic maturation through affecting ERα dynamic changes (Ding et al., 2020b). Of note, the same phenomenon was observed in estrogen-exposed oocytes. These results implied that EDCs may affect oocyte maturation by affecting ERα dynamic, very likely through the effects of ERα on spindle assembly. Regardless of its findings in mitosis, the role of ERα in the assembly of meiotic spindles is not very clear, and it will be a main content of our subsequent research. Altogether, these data led to speculate that BPB exposure induces dysfunction of ERα to disrupt mouse oocyte meiotic maturation *in vitro*.

Ovarian potential to generate viable oocytes is limited from puberty to menopause. In humans, immature oocytes are arrested at the diplotene stage of meiotic prophase I, and this dormancy continue for several decades (Holt et al., 2013). Long-term growth arrest increases the susceptibility to external stimuli for immature oocytes (Ge et al., 2019). For the potential impact of BPB exposure on oocyte meiotic maturation, PBE and cell cycle progression were explored in the BPB-exposed oocytes. Our findings concluded that BPB exposure compromised oocyte meiotic maturation and resulted in the meiotic arrest. The meiotic arrest is mainly due to the activation of the SAC induced by defective spindle morphology (Lara-Gonzalez et al., 2012; Ding et al., 2020a). The failure of meiotic maturation prompts us to detect the spindle organization and chromosome alignment in MI oocytes. In BPB-exposed oocytes, as shown by immunofluorescence results, disruption of the spindle structure and chromosome alignment were observed. Unlike mitosis, oocyte-meiotic spindles assembly without canonical centrosome function, but MTOCs contained centrosomal proteins for meiotic spindle organization (Lee et al., 2000; Meunier and Vernos, 2012). Given the aberrant spindle assembly, we also examined p-MAPK, a well-established MTOC-associated protein (Ding et al., 2017). The disrupted localization of p-MAPK in BPB-exposed oocytes suggested it contributed to defects of spindles. To avoid false chromosome separation, SAC is continuously activated in oocytes to prevent abnormal chromosome segregation to ensure proper meiotic maturation (Gorbsky, 2015). Our results suggested that the SAC was not deactivated after BPB exposure inducing the failure of meiotic metaphase to anaphase, which is another contributor to BPB-induced meiotic arrest. After chromosome separation, oocytes enter ATI stage and extrude PB1, and are arrested at MII stage waiting for fertilization. Any error in the meiotic process can lead to the failure of oocyte maturation, causing pregnancy loss and developmental disabilities in humans (Jones and Lane, 2013).

A previous study reported that BPB exposure induced DNA damage in sperm cells (Ullah et al., 2019). Consistently, BPB exposure resulted in DNA damage in oocytes as indicated by positive γ.H2A.X spot in our study. Pathways, DNA damage response can detect and repair the DNA (Winship et al., 2018). Initially, recovery of DNA damage in oocytes is mediated by DNA damage response, in case the DNA damage could not be repaired, it results in apoptosis (MacLennan et al., 2015). In addition to apoptosis, DNA damage if failed to repair immediately causes chromatin remodeling, cell cycle arrest, or cell cycle delay (Dasika et al., 1999; van Gent et al., 2001; Pandita and Richardson, 2009). Moreover, it was suggested that DNA damage induced meiotic arrest in mouse oocytes was intervened by the SAC (Collins et al., 2015). In our study, BPB exposure did not affect GVBD occurrence after 14 h of culture. Still, a large proportion of oocytes were arrested at MI stage with SAC activation, indicating that DNA damage may play a role in meiotic arrest induced by BPB exposure. EDCs can disrupt mitochondrial function and cause oxidative stress. Oxidative metabolism in mitochondria produces cellular ATP, as a side effect of oxidative phosphorylation, ROS are generated which could damage various biological macro-molecules (Yu et al., 2010; Kawamura et al., 2018). In particular, suitable ROS and ATP are crucial for proper spindle assembly in oocyte meiosis (Ding et al., 2020a). BPB has also been found to induce oxidative stress under *in vitro* conditions (Ullah et al., 2018a). We thus detected the ROS production after BPB treatment. Unexpectedly, BPB treatment did not lead to excessive ROS production, indicating oxidative stress was not produced by BPB. In consistent, the ATP levels were not changed by BPB exposure. These data suggested that mitochondrial function was not affected by BPB exposure.

Spatiotemporal gene expression is a prerequisite for oocyte maturation, and to a certain extent is achieved through epigenetic mechanisms. Epigenetic modifications in oocytes could be changed after exposure to BPA and its analogs. The expression of H3K4me2 and DNA methylation (5 mC) levels was altered after BPA treatment in porcine oocytes (Wang et al., 2016). Oocyte quality was also impaired by BHPF exposure through changing histone modifications, demonstrated by the increased H3K9me3 and H3K27me3 levels (Jiao et al., 2019). Any alteration in stage-dependent histone modifications can result in related to oocyte meiosis and quality. Notably, any induced epigenetic modification tends to be inherited to the next generation (Anway et al., 2005; Skinner et al., 2011). Based on our results, BPB exposure could change histone methylation (H3K9me3 and H3K27me3), suggesting that BPB could affect epigenetic modifications in mouse oocytes.

In conclusion, our results indicated that BPB exposure compromised oocyte meiotic maturation and damaged oocyte quality through affecting spindle assembly and chromosome alignment, acetylation of α-tubulin, DNA damage, epigenetic modifications, and ERα dynamics in mouse oocytes. Our study suggests that BPB may not be a safe alternative to BPA via an acute exposure *in vitro*. It warrants further studies to discourse the persistent impact of BPB chronic exposures at a low dose.

## Data Availability Statement

The original contributions presented in the study are included in the article/Supplementary Material, further inquiries can be directed to the corresponding author/s.

## Ethics Statement

The animal study was reviewed and approved by the Huazhong Agricultural University Animal Care and Use Committee (HZAUSW-2017-005).

## Author Contributions

L-JH and S-XZ conceived and designed the experiments. Z-MD performed the experiments. S-XZ, Z-MD, Y-SW, and Z-QD analyzed the data and participated in discussion. L-JH, S-XZ, J-JX, and Y-LM contributed reagents, materials, and analysis tools. S-XZ, Z-MD, and MA wrote and revised the manuscript. All authors contributed to the article and approved the submitted version.

## Conflict of Interest

The authors declare that the research was conducted in the absence of any commercial or financial relationships that could be construed as a potential conflict of interest.

## Supplementary Material

Supplementary Figure 1 BPB exposure disturbed the MII spindle assembly and chromosome alignment. **(A)** Images depicting spindle morphology and chromosome alignment in control and BPB-treated MII oocytes. α-tubulin, green; DNA, blue. Bar, 20 μm. **(B)** The rate of aberrant spindle morphology and chromosome alignment after BPB exposure. Control, *n* = 102; BPB, *n* = 91. ^∗∗^Significantly different (*P* < 0.01).
