# Supplementary figures and images for: Bisphenol B Exposure Disrupts Mouse Oocyte Meiotic Maturation in vitro Through Affecting Spindle Assembly and Chromosome Alignment
Source: Front Cell Dev Biol. 2020 Dec 17;8:616771. doi: 10.3389/fcell.2020.616771 (PMC7773771; doi:10.3389/fcell.2020.616771)

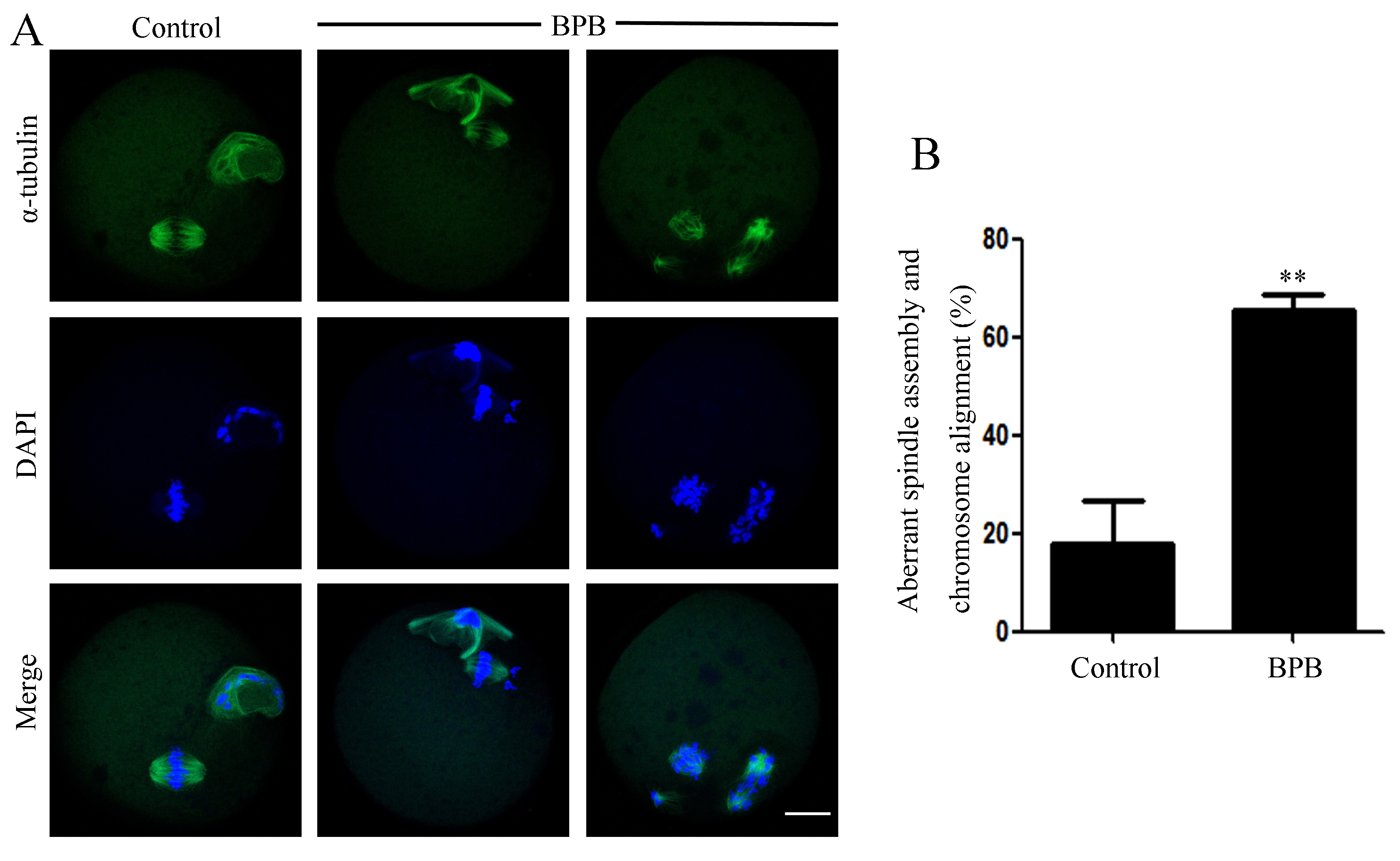

Supplement: Supplementary Figure 1 — BPB exposure disturbed the MII spindle assembly and chromosome alignment. (A) Images depicting spindle morphology and chromosome alignment in control and BPB-treated MII oocytes. α-tubulin, green; DNA, blue. Bar, 20 μm. (B) The rate of aberrant spindle morphology and chromosome alignment after BPB exposure. Control, n = 102; BPB, n = 91. ∗∗Significantly different (P < 0.01). [file Image_1.JPEG]
